# DRAGoN: a robust pipeline for analyzing DRUG-seq datasets

**DOI:** 10.1093/bioadv/vbaf214

**Published:** 2025-09-08

**Authors:** Scott Norton, John M Gaspar

**Affiliations:** Department of Data Science & Scientific Informatics, Research and Development Sciences—Information Technology, Merck & Co., Inc., Cambridge, MA, 02141, United States; Department of Data Science & Scientific Informatics, Research and Development Sciences—Information Technology, Merck & Co., Inc., Cambridge, MA, 02141, United States

## Abstract

**Motivation:**

Existing bioinformatics pipelines to process DRUG-seq datasets have limited flexibility and can have difficulty analyzing current datasets without requiring excessive computational time or memory.

**Results:**

Here, we describe an alternative, DRAGoN, which is fast, robust, and performs as well as or better than competing pipelines on key benchmarks without sacrificing accuracy. This is accomplished primarily via a preliminary demultiplexing step that facilitates the parallelization of the pipeline as well as the collection of per-well statistics that assist with quality control. DRAGoN provides the user maximum flexibility with respect to filtering, alignment, counting, and downsampling, and it efficiently collapses UMIs.

**Availability and implementation:**

DRAGoN is a Nextflow pipeline that utilizes open-source software alongside custom C++ programs and Python scripts. It is freely available at https://github.com/MSDLLCPapers/DRAGoN

## 1 Introduction

Digital RNA with pertUrbation of Genes (DRUG-seq) is a recent technique for enabling high-throughput whole transcriptome expression profiling of cultured cells in multiwell plates (Ye *et al.* 2018). In contrast to typical Bulk RNA-seq, DRUG-seq allows for the cost-effective study of the gene expression effects of small molecules, guide RNAs, and other perturbagens, in parallel and with replicates.

In DRUG-seq, the treated cells in multiwell plates are lysed, and reverse transcription is performed using oligos that include barcodes that are unique to each well. After paired-end sequencing, the R1 reads contain the well barcodes that can subsequently be used for demultiplexing, as well as unique molecular identifiers (UMIs) that allow for the removal of PCR duplicates. The R2 reads match the original mRNA sequence.

To produce a matrix of transcript counts, a computational pipeline for DRUG-seq must consist of several steps: QC filtering, aligning the R2 reads to a genome (or transcriptome), counting gene overlaps, demultiplexing the reads by the well barcodes, and removing PCR duplicates based on the UMIs. One such pipeline can be created from ST Pipeline (Navarro *et al.* 2017), which was designed for spatial transcriptomics but is easily adapted for DRUG-seq. Another pipeline is zUMIs (Parekh *et al.* 2018), which can perform this analysis from a list of known barcodes, such as in DRUG-seq. A third option is STARsolo (Kaminow *et al.* 2021), a convenient add-on to the STAR aligner that includes parsing of barcodes and UMIs as well as flexible handling of multimapping reads. An important consideration with these pipelines is scalability, as some of them can require excessive computational times and/or memory with larger DRUG-seq datasets.

Here, we introduce **D**RUG-seq/**R**NA-seq **A**nalysis of **G**enetically perturbed cells **o**n **N**extflow (DRAGoN), an open-source pipeline that efficiently processes DRUG-seq datasets. In contrast to the other pipelines, the first step of DRAGoN is to demultiplex the reads by well barcode, thus splitting the large datasets into separate bins that are then processed in parallel. This greatly limits the computational requirements of the pipeline and facilitates the collection of statistics per well throughout the rest of the pipeline, which is useful in troubleshooting. DRAGoN provides flexibility with respect to the alignment and counting steps, and it efficiently collapses UMIs via custom code. DRAGoN is wrapped in Nextflow (Di Tommaso *et al.* 2017) for robust execution.

## 2 Methods

### 2.1 DRAGoN workflow

The DRAGoN pipeline estimates gene expression for DRUG-seq experiments, or any other RNA-seq assay that includes barcodes and UMIs. Unlike other workflows which process the barcodes after alignment, DRAGoN demultiplexes as part of its initial QC processing. This step is implemented in a single custom C++ program, by default allowing up to one base substitution in the barcode. The program also filters and trims the reads in a manner similar to the ST Pipeline’s QC step. Wells with outlier read counts are flagged; the pipeline provides a variety of options for the downsampling of those wells using samtools view (Li *et al.* 2009) (Note 1, available as supplementary data at *Bioinformatics Advances* online).

After demultiplexing, DRAGoN processes each well’s reads in parallel. Demultiplexed reads are aligned to a reference genome using STAR 2.7.11b (Dobin *et al.* 2013, Kaminow *et al.* 2021) and assigned to gene features using featureCounts from subread 2.0.6 (Liao *et al.* 2014). Many of these tools’ configuration options are exposed directly as parameters to the Nextflow pipeline, with the remainder captured via an extra parameter that offers additional control over mapping and assignment behavior. Final UMI parsing and counts matrix generation are performed by a second custom C++ program. UMIs are collapsed if they share alignment coordinates with a 10 bp position tolerance and their sequences are no more than one edit distance apart. By default, only reads with a single gene assignment are counted (unique mappers); however, reads overlapping two or more genes (multimappers) can be distributed according to any one of four user-selected strategies, similar to those defined in STARsolo (Kaminow *et al.* 2021) (Note 1, available as supplementary data at *Bioinformatics Advances* online). At the end of the pipeline, sparse and dense counts matrices are generated, along with TPM and FPKM normalized counts, as the final outputs (Note 2, available as supplementary data at *Bioinformatics Advances* online).

**Figure 1.**
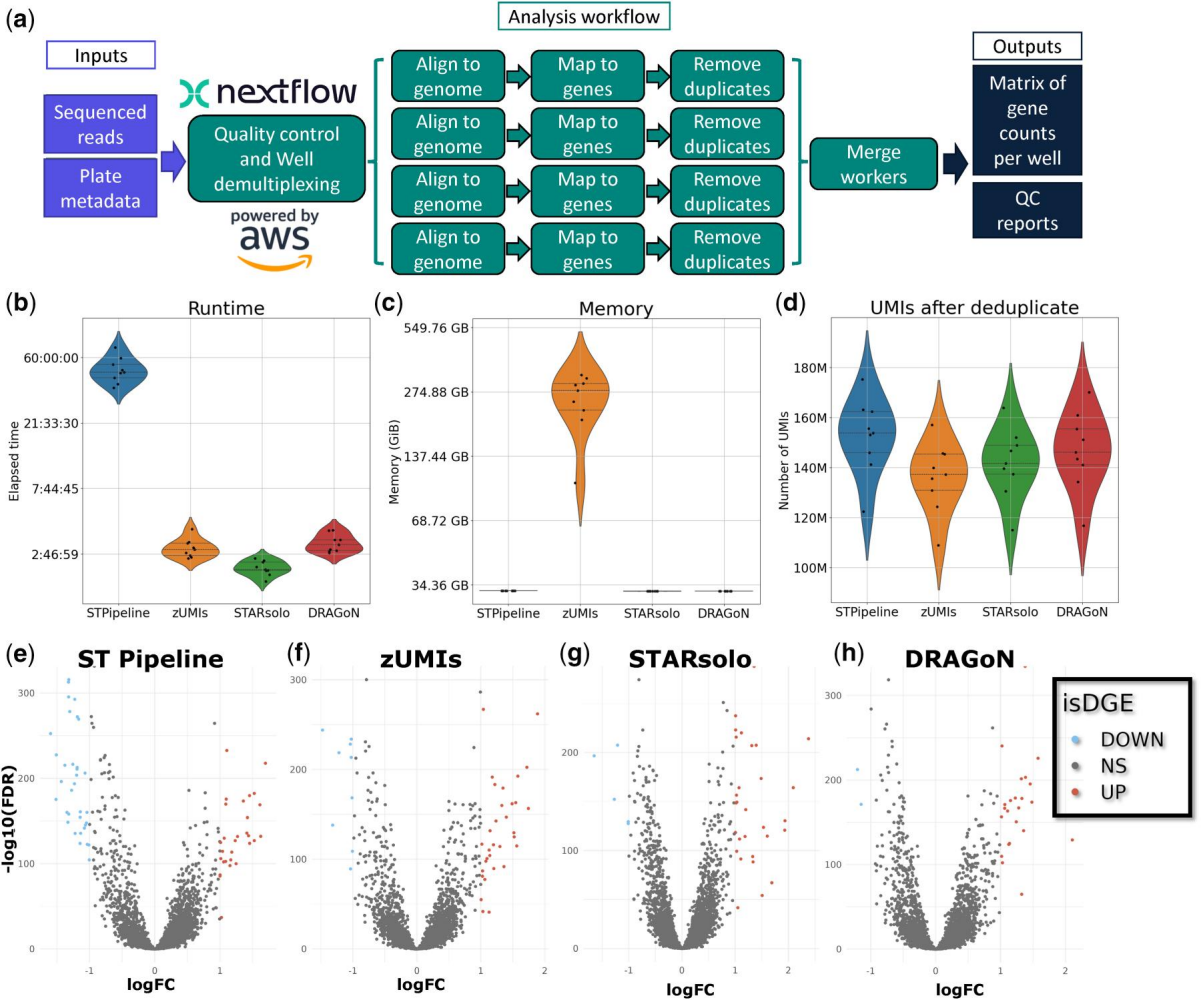
(a) Schematic of the DRAGoN pipeline. All processes are parallelized by plate, and the Align, Count, and Deduplication processes are further parallelized by well. (b–d) Violin plots comparing the total active runtime (b), peak memory usage (c), and total number of UMIs (d). Each violin is one pipeline, and each point is one plate. For memory and runtime, the vertical axis is presented on a natural logarithmic scale. *P*-values are unadjusted from Mann-Whitney pairwise tests. (e–h) Representative volcano plots comparing two replicate plates to each other based on output from STPipeline (e), zUMIs (f), STARsolo (g), and DRAGoN (h). Differentially expressed genes at |log2FC| > 1, FDR < 0.05 are indicated by DOWN or UP.

Because of the up-front barcode demultiplexing, DRAGoN is uniquely able to collect statistics for each well, and it does so at each step of the pipeline (QC, downsampling, alignment, feature assignment, and UMI parsing). This allows one to audit the results of the pipeline with some granularity and troubleshoot any irregularities that may arise (Note 2, available as supplementary data at *Bioinformatics Advances* online and Fig. 1, available as supplementary data at *Bioinformatics Advances* online). For instance, a low alignment rate may be due to an incorrect reference genome being used, but if it is observed only in certain wells, that may indicate sample contamination. As another example, excessive data loss at the UMI deduplication step may be due to overamplification, especially if it is observed disproportionately in wells with more reads at the beginning of the pipeline.

### 2.2 Benchmarking

DRAGoN v1.7.0 was benchmarked against ST Pipeline 1.7.6, zUMIs 2.9.7, and STARsolo 2.7.11b on a set of nine replicate plates from Novartis (Li *et al.* 2022), using the GRCh38 genome reference and Ensembl108 annotation. Each plate, pooled from 360 wells each, was sequenced to an average depth of 524 million reads. Each ST Pipeline and STARsolo job was run on a centos7-x86_64 Linux machine using Sun Grid Engine to allocate compute resources with 8 CPUs and 128 GB of RAM each. zUMIs was run on a dedicated research compute node with 8 CPUs and 750 GB of RAM. DRAGoN was launched in nextflow v24.10.6 with the JVM running in an r6i.large instance; resources for each process were allocated from a pool of r6i instances according to the pipeline’s configuration. Parameters used for each pipeline are described in Note 3, available as supplementary data at *Bioinformatics Advances* online. Per-plate runtime statistics were collected from the grid engine logs or from the “realtime” column in Nextflow’s trace file. For DRAGoN which processed wells individually, we selected the well for each plate with the worst performance on the runtime and memory metrics, discounting any time spent waiting for the batch scheduler. Multimapped reads were included in the final UMI counts. The respective outputs on plates VH02001612, VH02001614, and VH02001618 were compared against each other for between-plate, within-pipeline differential expression analysis using edgeR’s (v3.4.0, R v4.2.3) (Chen *et al.* 2025) glmQLFit and glmQLFTest methods with design formula ∼ Sample + plate_barcode + pipeline. Between-pipeline DGE was performed on the wells annotated as DMSO with design formula ∼ plate_barcode + pipeline. zUMIs counts were extracted from the $umicount$exon$all matrix in the dgecounts.rds output. Because zUMIs uniformly distributes multimapped reads across genes, DRAGoN and STARsolo were run with the Uniform multimap resolution strategy (Note 2, available as supplementary data at *Bioinformatics Advances* online). ST Pipeline reports ambiguously mapped reads as separate columns in its counts matrix; these were also uniformly distributed on-the-fly. Differentially expressed genes were identified as those with |log2FC| > 1 and Benjamini-Hochberg corrected FDR < 0.05.

## 3 Results and discussion

On each benchmark metric of runtime, memory, and final UMI counts, DRAGoN performed as well as or better than the alternative pipelines (Fig. 1b–d). Both DRAGoN and STARsolo overcome the major issues with ST Pipeline’s long runtime and zUMIs’ high memory requirements. ST Pipeline required 50 hours on average to process each plate (Fig. 1b), and zUMIs needed 300–500 GB to compute the UMI distance matrix (Fig. 1c). All four pipelines yielded similar UMI counts to each other (Fig. 1d). Although STARsolo finished 1.1 hours faster than DRAGoN, on average (Fig. 1b), it is important to note that DRAGoN spent an average of 2.9 hours on a separate QC step which STARsolo did not.

We tested the output matrices from each pipeline for differential gene expression between plates that were annotated as replicates of each other, with the expectation that few if any features would reach significance. Overall, ST Pipeline’s UMI counts resulted in the most differentially expressed genes (DEGs) between replicates, followed by zUMIs, DRAGoN, and STARsolo (Fig. 1e–h). DRAGoN’s LogFC estimates were most strongly correlated with STARsolo (Pearson *R*^2^ ∼ 0.96) followed by ST Pipeline (0.94), demonstrating DRAGoN’s quantitative consistency with established methods. By contrast, the lowest Pearson coefficients were observed between zUMIs and any other pipeline (Fig. 2, available as supplementary data at *Bioinformatics Advances* online). Average logCPM per gene correlated strongly between replicate plates for each pipeline (Fig. 3, available as supplementary data at *Bioinformatics Advances* online). Next, we tested for differentially expressed genes between pipelines on all the DMSO wells, again expecting very few DEGs to arise. The comparisons with the most DEGs all involved STARsolo (248 with zUMIs and 153 with ST Pipeline). The fewest DEGs were observed between DRAGoN and ST Pipeline (48) followed by between zUMIs and ST Pipeline (94) (Fig. 4, available as supplementary data at *Bioinformatics Advances* online), consistent with the logFC correlation observations.

In summary, DRAGoN offers a fast, accurate, and resource-distributed approach to DRUG-seq quantification. DRAGoN’s efficiency stems from distributing the workload across multiple compute instances, enabled by parsing the well barcodes as a first step. Up-front demultiplexing also facilitates the collection of QC and alignment statistics for each well independently; an output report file that compiles all these statistics can aid users in diagnosing whether unexpected results are biologically relevant or possible artifacts of the protocol. As a Nextflow pipeline, DRAGoN can be configured to take full advantage of any compute cluster or cloud environment.

## Supplementary Material

vbaf214_Supplementary_Data

## Data Availability

Source code for this project is available under the MIT license at https://github.com/MSDLLCPapers/DRAGoN. The data analyzed in this article were previously published (Li *et al.* 2022) and are available in the NCBI Gene Expression Omnibus (https://www.ncbi.nlm.nih.gov/geo/) at accession GSE176150.
