# Supplementary material for: DRAGoN: a robust pipeline for analyzing DRUG-seq datasets
Source: Bioinform Adv. 2025 Sep 8;5(1):vbaf214. doi: 10.1093/bioadv/vbaf214 (PMC12457737; doi:10.1093/bioadv/vbaf214)
Supplement: vbaf214_Supplementary_Data [file vbaf214_supplementary_data.docx]

**DRAGoN: a robust pipeline for analyzing DRUG-seq datasets**

Scott Norton, John M. Gaspar

Supplementary Information

# Contents

1. Supplementary Figures
   1. Figure S1: Plate visualization: heatmap of input read counts
   2. Figure S2: DGE logFC correlation between pipelines
   3. Figure S3: logCPM correlation between plates within pipeline
   4. Figure S4: DGE volcano plots between pipelines
2. Supplementary Note 1: DRAGoN implementation details
3. Supplementary Note 2: DRAGoN output files
4. Supplementary Note 3: Tool usage
   1. ST Pipeline
   2. zUMIs
   3. STARsolo
   4. DRAGoN
5. Supplementary References

## Supplementary Figures


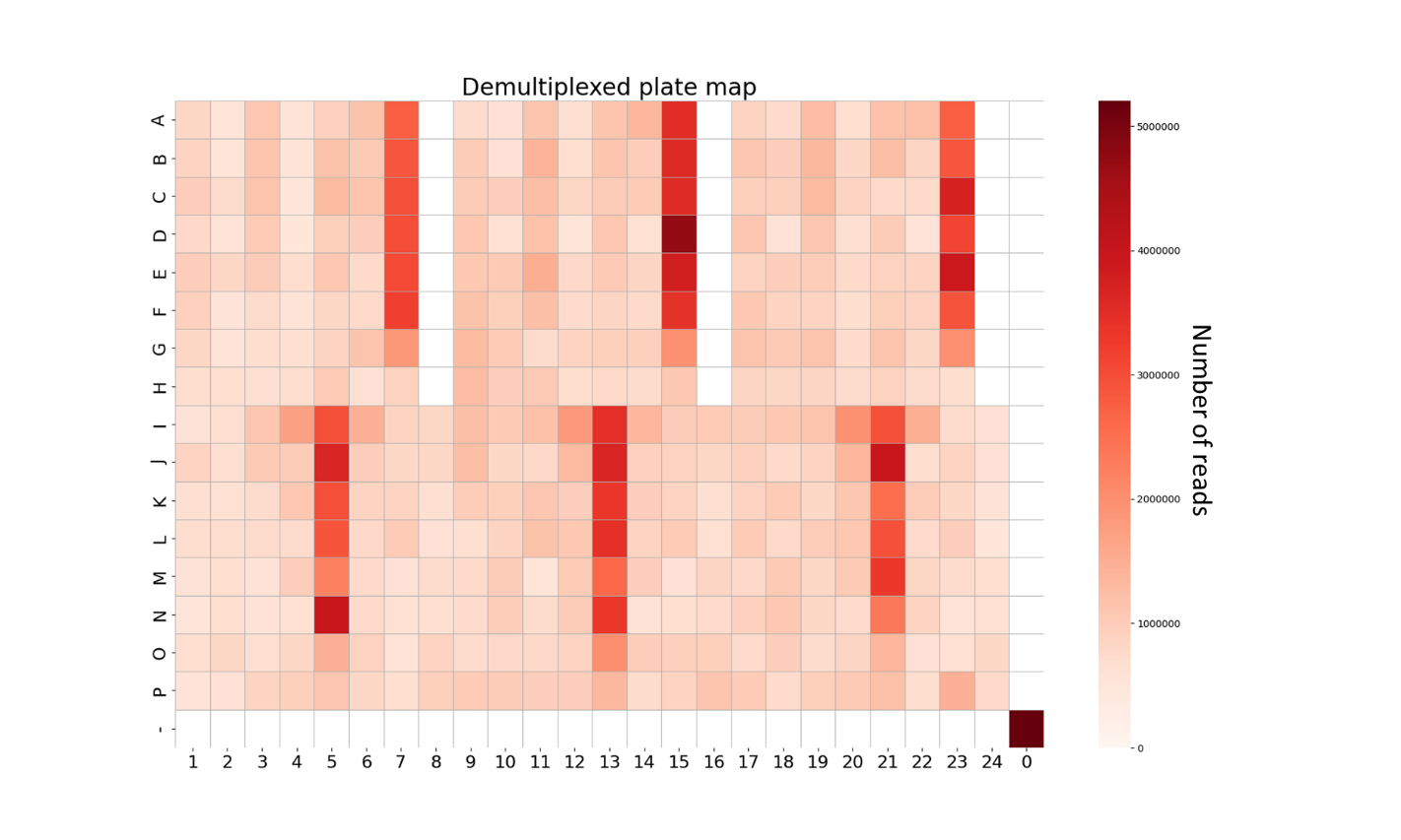


**Figure S1: Plate visualization: heatmap of input read counts**

Heatmap showing the number of input reads per sample well, arranged by the original plate layout. Deeper red indicates more reads. Blank cells correspond to unused barcodes. The bottom-right cell represents reads that do not match to any defined well barcode. We observe high read counts in wells treated with QC-05-UB63 (Homoharringtonine, wells I5-P5, I13-P13, and I21-P21) or KA-73-NB69 (Brusatol, wells A7-H7, A15-H15, and A23-H23). Wells A8-H8, A16-H16, and A24-H24 were empty, and wells I8-P8, I16-P16, and I24-P24 were DMSO controls.


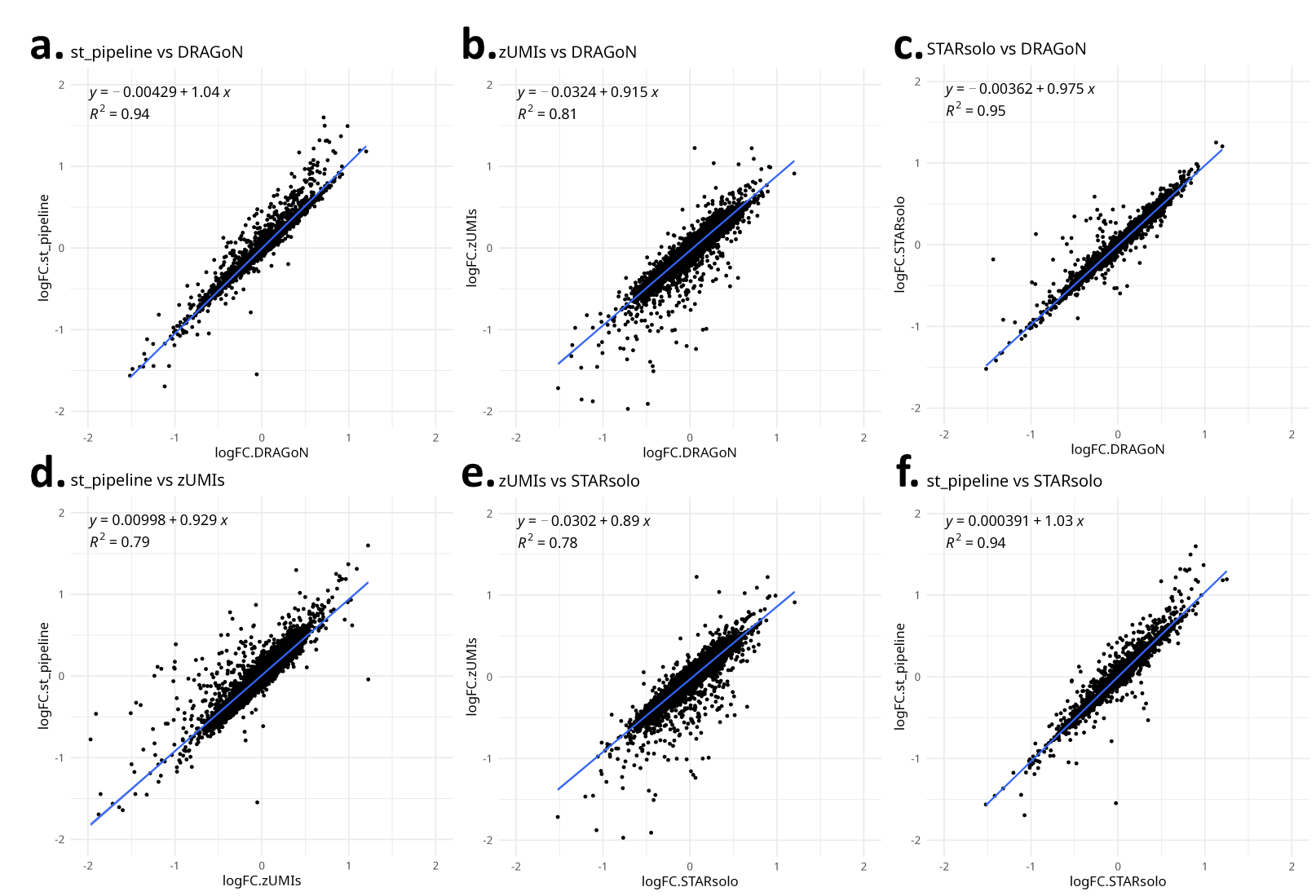


**Figure S2: DGE logFC correlation between pipelines**

Scatterplots comparing logFC estimates from the DGE tests in Fig. 1e-h. Trend lines and equations fit using a standard linear model. Correlation coefficient is the Pearson R^2^.


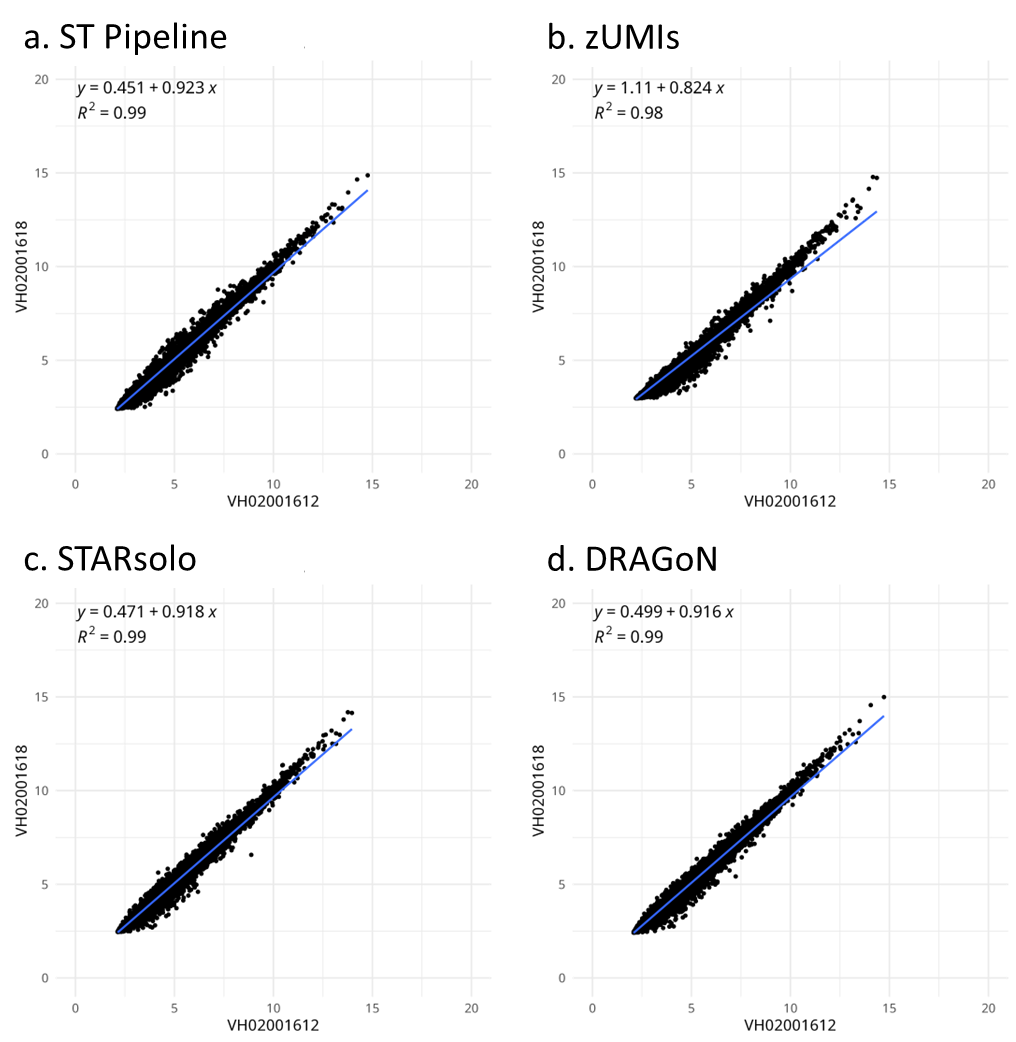


**Figure S3: logCPM correlation between plates within pipeline**

Average logCPM per gene was computed for each of VH02001618 and VH02001612 using the aveLogCPM method from edgeR. Each point represents one gene. The trendline, equation, and Pearson R^2^ represent the best linear fit between the two plates for each pipeline (a: ST Pipeline; b: zUMIs; c: STARsolo; d: DRAGoN).


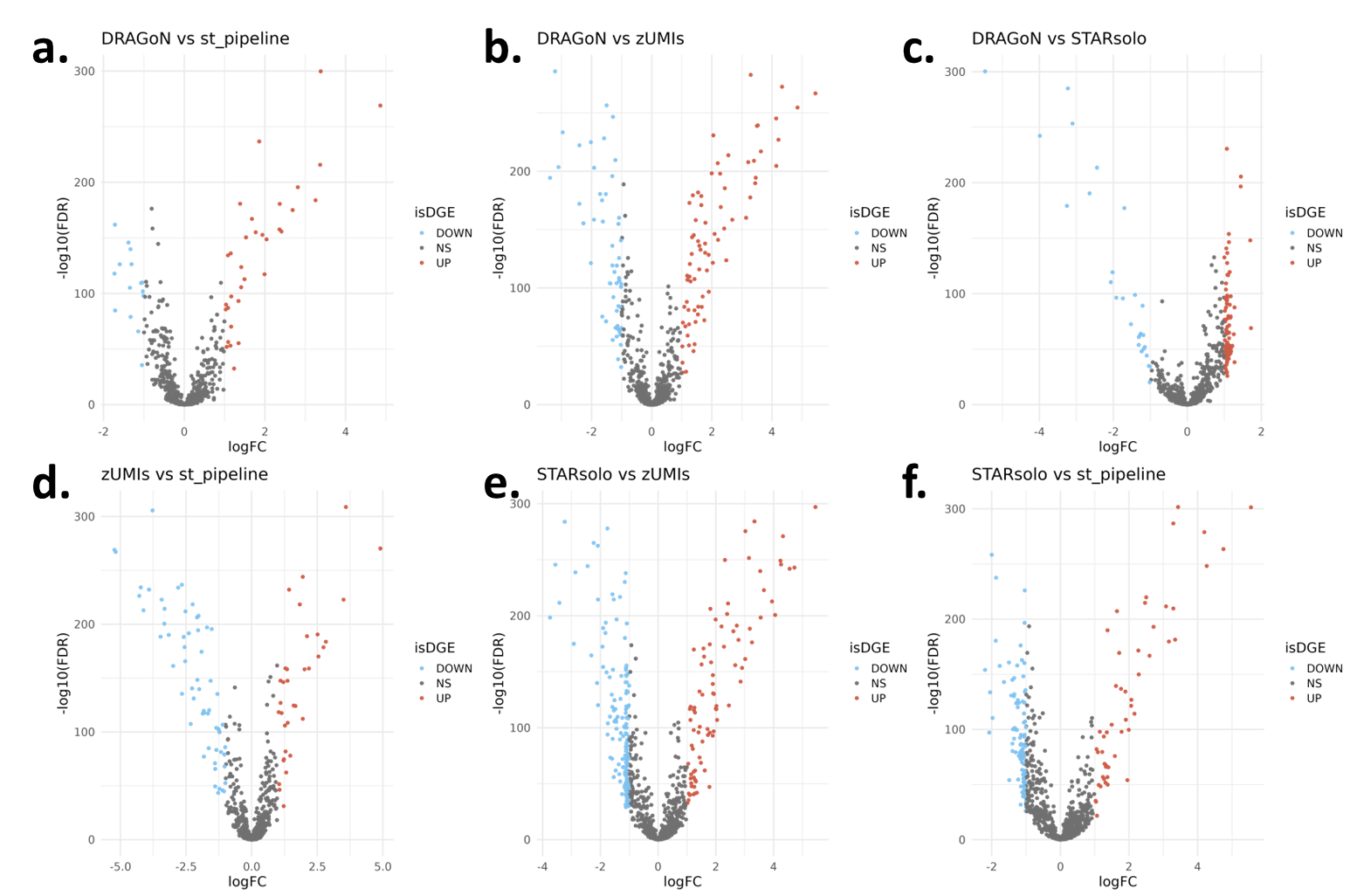
**Figure S4: DGE volcano plots between pipelines**

Representative volcano plots comparing each of the pipelines pairwise on their quantifications of the DMSO control wells.

**Supplementary Note 1: DRAGoN implementation details**

*QC filtering*

In the DRAGoN pipeline, reads are removed for low quality UMIs or excessive AT or GC content, and they are trimmed to remove adapters (via cutadapt [1]), homopolymers, and low quality regions. The reads are output into separate unmapped BAM files with the UMI and barcode sequences as SAM tags.

*Downsampling*

The UMI deduplication step of DRAGoN boasts memory efficiency and high accuracy at the cost of quadratic runtime scaling per well, similar to ST Pipeline (though the effect is lessened by distributing the workload per well). As a consequence, if a disproportionate amount of material comes from a small number of wells, those wells could require excessive computational time and thus delay the processing of the entire plate. The downsampling protocol was designed specifically to mitigate this sort of challenge.

When enabled, DRAGoN identifies wells with read counts above a threshold during the initial well demultiplexing step. By default, the threshold is 1.75 times the inter-quartile range above the 75th percentile, but one can specify a fixed number of reads (such as 5 million), or a proportion of the total number of reads, instead. For wells thus identified, DRAGoN uses samtools view to subsample the reads to the target maximum number. This maximum matches the threshold previously used, though again one can specify a fixed number or a proportion of the total number of reads.

*Feature assignment*

DRAGoN invokes featureCounts as its feature assignment method. By default, it runs in a relaxed mode that assigns reads to any feature it overlaps, even by one base. This facilitates counting unannotated splice isoforms as well as genes that overlap on the same strand. zUMIs also invokes the featureCounts algorithm directly using the rsubread package. By contrast, ST Pipeline imports the htseq counting methods and uses a strict overlap approach wherein all aligned positions must cover the feature for the gene to be counted. STARsolo takes a similar strict approach in line with the base aligner’s transcript quantification modes by default, so that its output is compatible with pseudocounters such as RSEM. However, one may specify the argument “--soloFeatures GeneFull” with STARsolo to amend this behavior and count reads spanning *de novo* splice junctions as well. DRAGoN does not force the user to subscribe to one philosophy or the other; rather it exposes parameters that grant control over featureCounts’ assignment logic, deferring the choice of how sensitive the workflow should be in this regard.

*Multimapping read resolution*

The DRAGoN pipeline allows the user to decide how to count reads overlapping two or more genes (multimappers). These strategies mirror those defined in STARsolo. The simplest strategy, “Uniform”, divides the count from a multimapper evenly across all overlapped features; this is similar to how zUMIs distributes multimapped reads. The “PropUnique” strategy divides a multimapper count based on the proportion of other reads that were uniquely assigned to each feature, falling back on Uniform when no reads map uniquely to these features. “Rescue” computes the harmonic mean of the Uniform and PropUnique estimates as an edge case correction for both strategies. Finally, “EM” uses expectation maximization to estimate the most likely distribution of a multimapper using the Rescue estimate as an initial state.

*Nextflow on AWS*

Because DRAGoN is implemented in Nextflow, it can be easily deployed on any compute environment. The benchmarks presented in this paper were performed using a cloud-based high-performance computing (HPC) cluster using Univa Grid Engine as a frontend to awsbatch queues. The runtime benchmark results presented for DRAGoN tracked only the critical path for the slowest well, but the delays in spinning up EC2 instances to handle the per-well division of labor resulted in an end-to-end runtime of about 9.5 hours. In production, DRAGoN is run in an AWS Batch compute environment with 8,192 max vCPUs and instance-type “optimal”. Under these conditions, DRAGoN still requires a full 10 hours to run end-to-end. Here the bottleneck stems from staging Nextflow process files between the S3 working bucket and the EFS mounted to the worker instance. MultiQC alone accounts for 2 hours 41 minutes of this runtime compared to just 3 minutes on the HPC, clearly demonstrating the high cost of network file transfers within AWS. A cloud filesystem such as Seqera FusionFS or FSX for Lustre can alleviate these slowdowns and any associated network and EC2 I/O costs [2]. Taking I/O overhead into account, the total compute time on AWS was closer to 7 hours. FusionFS is included as part of Seqera Platform [3], which was not evaluated as part of benchmarking.

**Supplementary Note 2: DRAGoN output files**

The DRAGoN pipeline has two primary outputs: the counts matrices, and the summary report.

*Counts Matrices*

DRAGoN outputs counts matrices in two formats: as a TSV file with row and column headers (counts.tsv), and as a MatrixMarket sparse matrix (DRAGoN.out/matrix.mtx), similar to the output from 10x Genomics’ cellranger program. This matrix tallies only the UMIs that were uniquely assigned to gene features. If the user selects one or more multimap handlers, for example “PropUnique”, those counts will be reported in separate output files. All of these matrices contain digital read counts suitable for differential expression analysis by a program such as edgeR. DRAGoN will also generate matrices that estimate fragments per kilobase per million UMIs (FPKM) and transcripts per million UMIs (TPM).

*Summary Report*

The output report file, DRAGoNreport.txt, is a tab-separated text file detailing QC statistics for each step in the pipeline, one row per well plus a row for reads that could not be assigned to a well. The first seven columns describe the initial read counts for each well, the percent of reads that were discarded by each processing step (QC, downsample, alignment, feature assignment, and UMI parsing), and the final UMI count. These columns can highlight serious problems in the data or pipeline parameters. For instance, a low alignment rate can indicate either sample contamination or that the wrong reference genome was used. High genome alignment rates followed by poor gene assignment rates may be indicative of an inappropriate transcriptome reference. Excessive data loss at the UMI deduplication step may be due to overamplification and/or a low amount of intact DNA at the beginning. A common occurrence that we have observed is a disproportionate number of reads being flagged as not matching a barcode at the initial demultiplexing step, which was indicative of an incorrect set of well barcodes being supplied to the pipeline.

A major advantage of reporting QC statistics per well is that it enables visualization in the same layout as the physical plate. DRAGoN produces two summary-level plots as a byproduct of the QC workflow to allow inspection of read distribution among the set of expected barcodes (see Fig. S1).

The remaining columns in the report provide additional details about how many reads passed or failed each QC filtering criterion. Detailed alignment stats are included in the MultiQC report generated at the end of the pipeline. This report also includes pre- and post-filtering FastQC reports, though on a per-plate basis rather than per-well.

**Supplementary Note 3: Tool usage**

This section details how each pipeline was invoked for the benchmark tests. The input for all pipelines includes a STAR index (versionGenome=2.7.4a) and corresponding GTF annotation, paired-end gzipped fastq files obtained using the NCBI SRA toolkit, and [GSE176150_metadata.csv.gz](https://www.ncbi.nlm.nih.gov/geo/query/acc.cgi?acc=GSE176150) (the metadata table). The metadata table was sorted by “plate_barcode” and “plate_well”, then augmented with a unique well identifier “well_name” which is the concatenation of “plate_barcode”, “plate_well”, and “Sample”.

*ST Pipeline*

The metadata table was split by “plate_barcode”, and colums “well_index” and “well_name” were pulled into a TSV file for each plate. Dummy coordinate columns were added to satisfy ST Pipeline’s requirements. The result was saved as barcodes.txt.

st_pipeline_run.py --ids barcodes.txt --ref-map <STAR-index> --ref-annotation <GTF-file> --expName <PlateName> --output-folder <OutputPath> --temp-folder $(mktemp -d) --start-id 0 --umi-start-position <BarcodeLength> --umi-end-position <R1Length> --umi-counting-offset 250 --allow-missed 1 --log-file st_pipeline.log --verbose R1.fastq.gz R2.fastq.gz

*zUMIs*

zUMIs run parameters are specified via a YAML file. The zUMIs package includes a template defining defaults and indicating parameters which are required. The template was augmented with the following parameters. Here barcodes.txt contains only the “well_index” and “well_name” columns.

project: <PlateName>

sequence_files:

file1:

name: R1.fastq.gz

base_definition:

- BC(1-<BarcodeLength>)
- UMI(<BarcodeLength+1>-<R1Length>)

file2:

name: R2.fastq.gz

base_definition:

- cDNA(1-<cDNAlength>)

reference:

STAR_index: <STAR-index>

GTF_file: <GTF-file>

out_dir: <OutputPath>

barcodes:

barcode_file: barcodes.txt

nReadsPerCel: 0

automatic: no

counting_opts:

strand: 1

Ham_Dist: 0

multi_overlap: yes

twoPass: no

downsampling: “10000-20000”

introns: no

mem_limit: 64

num_threads: 8

*STARsolo*

STARsolo’s parameters are derived from ST Pipeline’s invocation with additional Solo parameters. Here barcodes.txt contains only the “well_index” column.

STAR --runThreadN 8 \

--readFilesIn R2.fastq.gz R1.fastq.gz \

--outFileNamePrefix <PlateName> \

--readFilesCommand pigz -cd \

--genomeDir <STAR-index> \

--sjdbGTFfile <GTF-file> \

--outFilterType Normal \

--outFilterMultimapNmax 20 \

--outFilterMultimapScoreRange 1 \

--alignIntronMin 1 \

--alignIntronMax 1 \

--soloType CB_UMI_Simple \

--soloCBlen <BarcodeLength> \

--soloUMIstart <BarcodeLength+1> \

--soloUMIlen <R1Length – BarcodeLength> \

--soloCBwhitelist <barcodes.txt> \

--soloCBmatchWLtype 1MM_multi_Nbase_pseudocounts \

--soloCellFilter None \

--soloMultiMappers Uniform \

--outSAMtype BAM SortedByCoordinate \

--outBAMsortingThreadN 1 \

--limitBAMsortRAM 68000000000 \

--outMultimapperOrder Random \

--outSAMattributes NH HI nM AS CR UR CY UY CB UB GX GN sS sQ sM \

--outFilterMatchNmin 20 \

--outFilterMismatchNoverLmax 0.1 \

--outSAMorder Paired \

--outSAMprimaryFlag OneBestScore \

--outSAMunmapped Within \

--clip5pNbases 0 \

--clip3pNbases 0 \

--alignEndsType Local \

--soloFeatures GeneFull

*DRAGoN*

Instead of a text file, DRAGoN expects the metadata to be an Excel spreadsheet. Each sheet represents one plate, and the columns are mapped as such:

index 🡪 Pool_Index_i7
 plate_well 🡪 Well_Index
 well_index 🡪 WellBarcode
 Sample 🡪 SampleName
 plate_barcode 🡪 Plate & PoolName
 well_name 🡪 SampleID

DRAGoN’s implementation is roughly equivalent to the following call chain:

DRAGoN/bin/metadata_to_run_specs.py metadata.xlsx [-s sampleSheet.csv]

# output: <PlateName>_barcodes.txt

fastqc -o <PlateName> --extract -f fastq -t <CPUs> --memory <RAM> --casava R1.fastq.gz R2.fastq.gz

DRAGoN/bin/demultiplex R1.fastq.gz R2.fastq.gz <PlateName>_barcodes.txt <PlateName> --cpus <CPUs> --bclen <BarcodeLength> --umistart <BarcodeLength + 1> --umilen <R1Length – BarcodeLength> --mismatch 1 --min-length 20 --min-qual 20 --min-qual-bases 6 --max-AT 0.9 --max-GC 0.9 --min-homop-len 10 --max-homop-mmatch 0 --max-bc-ns 1 --adapter CTGTCTCTTATA

# output: <PlateName>_unmap.bam, <PlateName>.demux.json

samtools split -@ <CPUs> -f <PlateName>_unmap_%!.%. -M <NumBarcodes + 1> -v -d BI <PlateName>_unmap.bam

# output: <PlateName>_unmap_<bcIdx>.bam, ...

# following steps are submitted per bcIdx

DRAGoN/bin/downsample_bam.py -I <PlateName>_unmap_<bcIdx>.bam -b <PlateName>_barcodes.txt -n <bcIdx> -s <PlateName> -p <PlateName>_<bcIdx> -d <PlateName>.demux.json -c <CPUs>

# output: <PlateName>_<bcIdx>_keep.bam, <PlateName>_<bcIdx>.downsample.txt

STAR --runThreadN <CPUs> --readFilesIn <PlateName>_<bcIdx>_keep.bam --outFileNamePrefix <PlateName>_<bcIdx>_ --genomeDir <STAR-index> --readFilesType SAM SE --readFilesCommand samtools view --outFilterType Normal --outFilterMultimapNmax 20 --outFilterMultimapScoreRange 1 --alignIntronMin 1 --alignIntronMax 1 --outSAMtype BAM Unsorted --outSAMattributes All --outFilterMatchNmin 20 --outFilterMismatchNoverLmax 0.1 --outSAMorder Paired --outSAMprimaryFlag OneBestScore --outMultimapperOrder Random --outSAMunmapped None --outReadsUnmapped Fastx --clip5pNbases 0 --clip3pNbases 0 –alignEndsType Local

# output: <PlateName>_<bcIdx>_Aligned.out.bam, <PlateName>_<bcIdx>_Unmapped.out.mate1

(samtools view -H <PlateName>_<bcIdx>_keep.bam; samtools import -T ‘*’ -0 <PlateName>_<bcIdx>_Unmapped.out.mate1) | samtools view -b -@ <CPUS> -o <PlateName>_<bcIdx>_Unmapped.out.bam

featureCounts -s 1 –fracOverlap 0 -a <GTF-file> -F GTF -M -O --fraction -T <CPUs> -R BAM -o <PlateName>_<bcIdx>.out

# output: <PlateName>_<bcIdx>.out.featureCounts.bam

samtools sort -n -T CB -O BAM -m <RAM> -@ <CPUs> -o <PlateName>_<bcIdx>.sortedByName.bam

DRAGoN/bin/deduplicate <PlateName>_<bcIdx>.sortedByName.bam <PlateName>_barcodes.txt <GTF-file> DRAGoN.out.<PlateName>_<bcIdx>/ --cpus <CPUs> --umilen <R1Length – BarcodeLength> --max-distance 1 --max-lookback 10 --mmap-strategies Uniform

# collect DRAGoN.out.* and *_Unmapped.out.bam, groupTuple by PlateName

samtools cat -@ <CPUs> <PlateName>_*_Unmapped.out.bam | samtools view -F QCFAIL | grep -oE ‘BI:Z:\w+’ | sort | uniq -c | sed -r ‘s/^\s*([0-9]+)\s+BI:Z:(\w+)/\2\t\1/g’ > <PlateName>_unmapped.txt

DRAGoN/bin/dragon_report.py --matrices DRAGoN.out.<PlateName>_* --dmux <PlateName>.demux.json --features <PlateName>_*.out.summary --downsample-report <PlateName>_*.downsample.txt –unmapped-report <PlateName>_unmapped.txt

# output: DRAGoN.out/, counts.tsv, counts-Uniform.tsv, DRAGoNreport.txt

The following software versions were used at runtime: java-openjdk-23.0.1 nextflow-24.10.6 samtools-1.21 STAR-2.7.11b subread-2.0.6 fastqc-0.12.1 multiqc-1.19 gawk-5.1.0.

C++ utilities were compiled by g++-13.2.0 in the context of cmake-3.29.2 and pkg-config-0.29.2 with the following libraries: eigen-3.2 bamtools-2.5.2 zlib-1.2.13 nlohmann_json-3.11.3 kseq++-1.1.2 bamtools-2.5.2 boost-1.84.0.

Python versions varied by process due to the disparate availability of suitable mulled images in biocontainers.

- S3 glacier restore uses python 3.12.2 with aioboto3==12.0.0.
- Metadata parsing uses python 3.12.9 using pandas==2.2.1, openpyxl==3.1.5.
- Well overenrichment reports are generated using python 3.12.5 with matplotlib==3.9.1, seaborn==0.13.2, pandas==2.2.2.
- Downsampling use python 3.9.13 with pysam==0.19.1, pandas==1.4.2.
- Report generation use python 3.13.2 with pandas==2.2.3, scipy==1.14.1.
- FPKM and TPM normalization uses python 3.13.2 with pandas==2.2.3.

Supplementary References

1. M. Martin, "Cutadapt Removes Adapter Sequences From High-Throughput Sequencing Reads," EMBnet, vol. 17, no. 1, pp. 10-12, May 2011.
2. P. Di Tommaso, "Selecting the right storage architecture for your Nextflow pipelines," 4 May 2023. [Online]. Available: https://www.nextflow.io/blog/2023/selecting-the-right-storage-architecture-for-your-nextflow-pipelines.html. [Accessed 5 September 2024].
3. Seqera, "Platform," Seqera, 2024. [Online]. Available: https://seqera.io/platform/. [Accessed 11 September 2024].
